# Insights Into Highly Improved Solar-Driven Photocatalytic Oxygen Evolution Over Integrated Ag_3_PO_4_/MoS_2_ Heterostructures

**DOI:** 10.3389/fchem.2018.00123

**Published:** 2018-04-18

**Authors:** Xingkai Cui, Xiaofei Yang, Xiaozhai Xian, Lin Tian, Hua Tang, Qinqin Liu

**Affiliations:** ^1^School of Materials Science and Engineering, Jiangsu University, Zhenjiang, China; ^2^College of Science, Nanjing Forestry University, Nanjing, China; ^3^State Key Laboratory of Photocatalysis on Energy and Environment, Fuzhou University, Fuzhou, China

**Keywords:** Ag_3_PO_4_, MoS_2_, composite photocatalyst, oxygen evolution, water splitting, Z-scheme

## Abstract

Oxygen evolution has been considered as the rate-determining step in photocatalytic water splitting due to its sluggish four-electron half-reaction rate, the development of oxygen-evolving photocatalysts with well-defined morphologies and superior interfacial contact is highly important for achieving high-performance solar water splitting. Herein, we report the fabrication of Ag_3_PO_4_/MoS_2_ nanocomposites and, for the first time, their use in photocatalytic water splitting into oxygen under LED light illumination. Ag_3_PO_4_ nanoparticles were found to be anchored evenly on the surface of MoS_2_ nanosheets, confirming an efficient hybridization of two semiconductor materials. A maximum oxygen-generating rate of 201.6 μmol · L^−1^ · g^−1^ · h^−1^ was determined when 200 mg MoS_2_ nanosheets were incorporated into Ag_3_PO_4_ nanoparticles, which is around 5 times higher than that of bulk Ag_3_PO_4_. Obvious enhancements in light-harvesting property, as well as electron-hole separation and charge transportation are revealed by the combination of different characterizations. ESR analysis verified that more active oxygen-containing radicals generate over illuminated Ag_3_PO_4_/MoS_2_ composite photocatalysts rather than irradiated Ag_3_PO_4_. The improvement in oxygen evolution performance of Ag_3_PO_4_/MoS_2_ composite photocatalysts is ascribed to wide spectra response in the visible-light region, more efficient charge separation, and enhanced oxidation capacity in the valence band (VB). This study provides new insights into the design and development of novel composite photocatalytic materials for solar-to-fuel conversion.

## Introduction

Inspired by natural photosynthesis, the construction of visible-light-responsive functional semiconducting materials for highly efficient photocatalytic water splitting and reduction of CO_2_ has drawn considerable attention over the past few years (Maeda and Domen, 2010; Mikkelsen et al., 2010; Takanabe, 2017; Zheng et al., 2017). Especially, water splitting into hydrogen and oxygen has been intensively investigated due to the nature of clean and sustainable solar-to-fuel conversion. Compared with the hydrogen evolution reaction (HER), four-electron water oxidation process is more difficult to be fulfilled since a higher potential more than 1.23 eV is required (Kudo and Miseki, 2009), mostly an overpotential is also required. Thus, oxygen evolution is considered as the rate-determining step in photocatalytic overall water splitting process. So far only few semiconductors such as WO_3_, BiVO_4_, have been employed as photocatalysts for oxygen production from water splitting (Xin et al., 2009; Wu et al., 2016; Zeng et al., 2017; He et al., 2018). Due to the limitations of band structures and light-harvesting properties in the visible light region, the utilization of sing-component semiconductors as catalysts for oxygen evolution has encountered serious difficulties, the design and development of novel composite materials for solar-driven photocatalytic water splitting are highly desirable.

Since the pioneer work of silver phosphate (Ag_3_PO_4_) semiconductor for photocatalytic applications in 2010 (Yi et al., 2010). Many efforts have been devoted to synthesize Ag_3_PO_4_ photocatalysts with different nanostructures and a variety of Ag_3_PO_4_-based composite photocatalytic materials for energy and environmental applications (Bi et al., 2012; Wang et al., 2012; Hu et al., 2013; Cao et al., 2017). Nanostructure engineering of pristine Ag_3_PO_4_ and hybridization of Ag_3_PO_4_ with other semiconductors have been proven to offer superior advantages in light absorption, electron-hole separation and charge transportation, resulting in the enhancement in the photocatalytic activity (Yang et al., 2015b; Lv et al., 2016; Wang et al., 2017; Zhou et al., 2018). The key to synthesizing highly efficient Ag_3_PO_4_-based composite photocatalysts lies in screening promising candidates with matched band structures and constructing heterojunctions with optimal morphologies and interfaces, where favorable visible light utilization and tandem charge transfer pathway should be taken into consideration. The past decades have witnessed the use of molybdenum disulfide (MoS_2_), a 2D lamellar material with excellent conductive property, as electrocatalysts and photocatalysts for applications in electrochemical and solar-to-fuel conversion (Xiang et al., 2012; Dai et al., 2017; Yang et al., 2017). It was confirmed that the combination of MoS_2_ with functional semiconductors enabled obvious enhancements in both photocatalytic and electrocatalytic hydrogen production activity (Sun et al., 2016; Zhang et al., 2016; Iqbal et al., 2017; Yuan et al., 2017). The hybridization of MoS_2_ with Ag_3_PO_4_ to produce MoS_2_/Ag_3_PO_4_ composite materials has been primarily explored, however the application is restricted to the photodegradation of different kinds of organic pollutants (Wang L. et al., 2015; Wang P. F. et al., 2015; Gyawali and Lee, 2016; Li et al., 2016; Zhu et al., 2016; Wan et al., 2017), the employment of MoS_2_/Ag_3_PO_4_ nanocomposites as photocatalysts for solar-light-driven oxygen evolution from water splitting has not yet been explored.

Most recently, we reported the *in-situ* deposition of Ag_3_PO_4_ on graphitic carbon nitride (g-C_3_N_4_) nanostructures for highly efficient Z-scheme oxygen evolution from water splitting (Yang et al., 2015a,c; Cui et al., 2018; Tian et al., 2018). In consideration of the matched band structures of bulk Ag_3_PO_4_ and MoS_2_ materials for redox reactions, in this work, we demonstrate the hybridization of oxygen-producing photocatalyst Ag_3_PO_4_ with few-layered, two-dimensional MoS_2_ nanosheets, and the use of Ag_3_PO_4_/MoS_2_ nanocomposites for photocatalytic water oxidation under LED illumination. As-prepared hybrid materials exhibit well-organized nanostructures, in which sheet-like MoS_2_ materials provide sufficient active sites for the deposition of Ag_3_PO_4_ nanoparticles. It is for the first time that oxygen evolution performance over the obtained Ag_3_PO_4_/MoS_2_ composite photocatalysts has been evaluated, moreover, the effects of highly conductive MoS_2_ materials on visible light utilization, electron-hole separation and water oxidation efficiency are systematically revealed.

## Experimental

### Preparation

MoS_2_ nanosheets were fabricated by the ultrasonic stripping of commercially available MoS_2_ materials. In a typical synthesis of Ag_3_PO_4_/MoS_2_ nanocomposites, different amounts of sheet-like MoS_2_ (50, 100, 200, 300 mg) were added into 90 mL H_2_O, respectively, followed by the ultrasonic treatment for 3 h. Next, 30 mL of AgNO_3_ (18 mmol, 3.06 g) aqueous solution was added dropwise into the MoS_2_ suspension, and stirred for further 12 h. And then 30 mL of Na_3_PO_4_ (6 mmol, 2.28 g) aqueous solution was added slowly into the above mixture, followed by continuous stirring for 3 h. After high-speed centrifugation, solid products were washed with deionized water and ethanol repeatedly, and dried at 60°C for 12 h. The final products were collected and are denoted as AM-50, AM-100, AM-200, and AM-300.

### Characterizations

X-ray diffraction (XRD) was measured using Cu Kα radiation with the 2θ range from 5 to 80° at a scan rate of 5° min^−1^ on D/MAX2500PC. Raman spectra were recorded by Thermo Scientific™ DXR spectrometer operating at 532 nm. X-ray photoelectron spectroscopy (XPS) was evaluated by Perkin-Elmer PHI 5000C. The field-emission scanning electron microscopy (FE-SEM) was performed on JSM-7001F. The Ultraviolet-visible diffuse reflectance spectrophotometer (UV-vis DRS) on UV2450 from 200 to 800 nm with BaSO_4_ as reference standard. Photoluminescence (PL) emission measurements were carried out by a QuantaMaster™ 40 with an excitation wavelength of 420 nm. The electron spin resonance (ESR) measurements were recorded on a JES FA200 Spectrometer using the 5, 5-dimethyl-1-pyrroline-N-oxide (DMPO) as the radical capture reagent.

### Photocatalytic measurements

The efficiency of photocatalytic oxygen evolution was monitored by *in-situ* oxygen sensor in a sealed system connected with a circulation system. Before the measurement, oxygen-free and air-saturated water were used to calibrate the oxygen probe with temperature compensation. For the measurement of oxygen evolution, 0.3 g of the photocatalyst powder was added into AgNO_3_ aqueous solution (100 mL, 10 g/L), followed by an ultrasonic treatment for 5 min.

## Results and discussion

**Figure 1:**
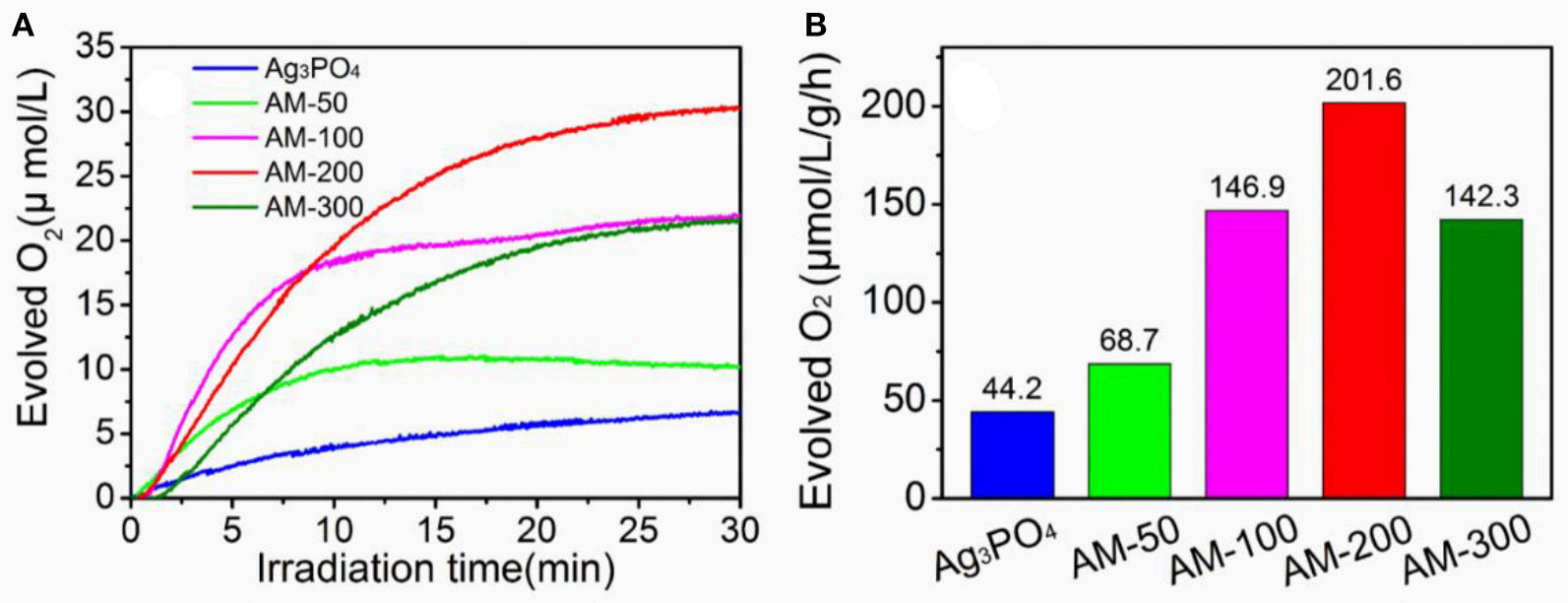
Oxygen-evolving concentrations **(A)** and rates **(B)** over different photocatalysts under LED illumination.

Photocatalytic oxygen evolution from water splitting over pure Ag_3_PO_4_ and Ag_3_PO_4_/MoS_2_ composites with different mass ratios were evaluated, and the results are presented in Figure 1. It can be observed (Figure 1A) that the amount of evolved oxygen increases gradually when 50 and 100 mg MoS_2_ were employed, the highest concentration of produced oxygen was recorded when the content of MoS_2_ was increased to 200 mg. Further increase in the MoS_2_ content from 200 to 300 mg in the Ag_3_PO_4_/MoS_2_ composite resulted in the deterioration in the oxygen-generating performance. Notably all the composites showed improved oxygen-evolving performance than bulk Ag_3_PO_4_ material. The oxygen-evolving rates of as-prepared samples under LED illumination are further quantified and shown in Figure 1B. When 200 mg MoS_2_ was introduced to hybridize with Ag_3_PO_4_, an optimal oxygen-generating rate of 201.6 μmol · L^−1^ · g^−1^ · h^−1^ was determined, which is about 4.5 times higher than that of pure Ag_3_PO_4_. It can be concluded from the oxygen evolution performance that a proper addition of MoS_2_ may promote the water oxidation efficiency, while the use of more than 200 mg of MoS_2_ is found to show negative effects on the oxygen evolution performance. For simplicity, only the composite AM-200 with the best oxygen-producing efficiency is chosen for comparisons with two starting materials in the following sections.

**Figure 2:**
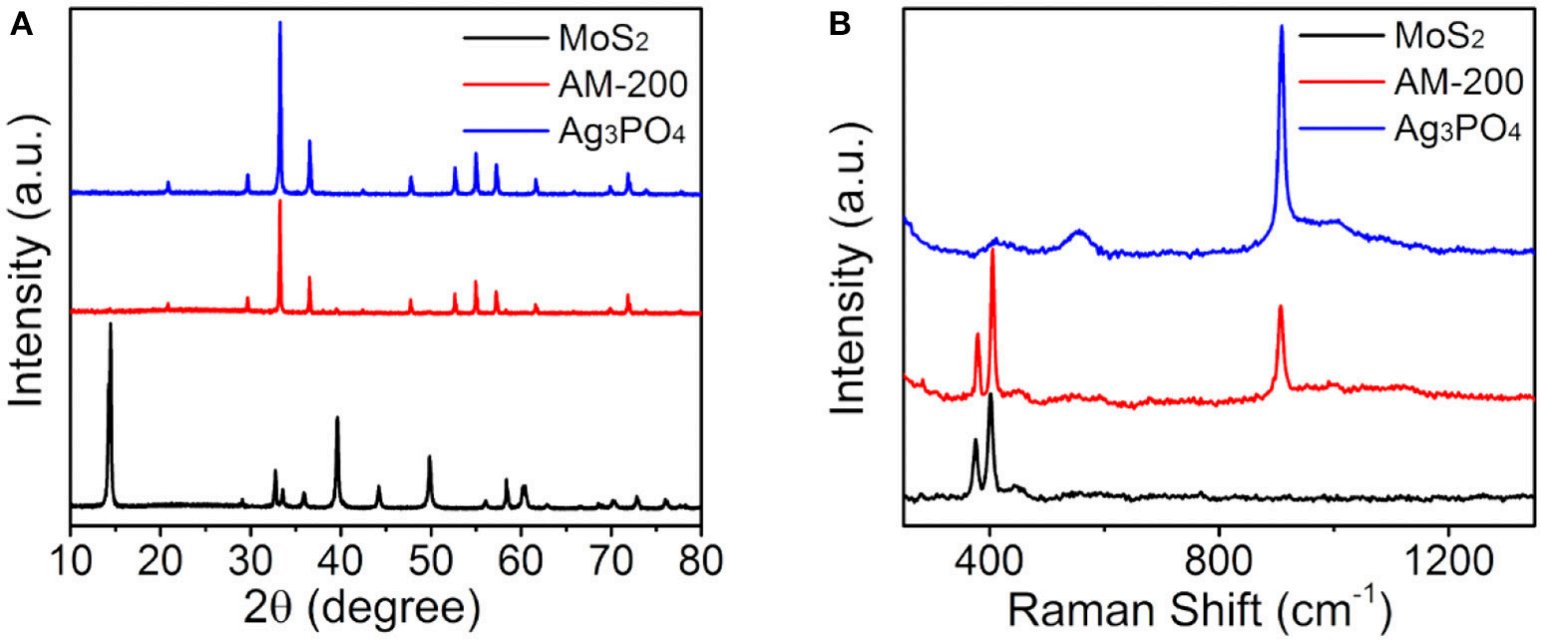
XRD patterns **(A)** and Raman spectra **(B)** of pure Ag_3_PO_4_, MoS_2_, and the composite AM-200.

Following the evaluation of photocatalytic oxygen evolution performance, phase structures of the AM-200 composite were confirmed by XRD patterns (Figure 2A). Diffraction peaks (black line) appearing in 14.32, 32.62, 33.44, 35,82, 39.48, 44,1, 49.72, 58.24, 60.32, and 72.72° can be assigned to the (002), (100), (101), (102), (103), (006), (105), (110), (008), and (203) planes of hexagonal MoS_2_ (JCPDS No. 37-1492). And the characteristic peaks (blue line) located at 20.96, 29.78, 33.38, 36.66, 47.86, 52.76, 55.1, 57.34, 61.72, and 73.94° correspond to planes (110), (200), (210), (211), (310), (222), (320), (321), (400), and (332) of bulk Ag_3_PO_4_ (JCPDS No. 06-0505), respectively. No obvious difference can be observed in characteristic diffraction peaks of bulk Ag_3_PO_4_ and Ag_3_PO_4_/MoS_2_ composite (AM-200), presumably due to a relatively weaker diffraction intensity of MoS_2_. The molecular structures of pure MoS_2_, bulk Ag_3_PO_4_ and the Ag_3_PO_4_/MoS_2_ composite AM-200 were further characterized by Raman spectra (Figure 2B). In the spectrum of Ag_3_PO_4_, a sharp absorption peak at 908.5 cm^−1^ can be attributed to the motion of terminal oxygen bond vibration in phosphate chains. The peak at 1002.4 cm^−1^ is ascribed to the asymmetric stretching vibrations of O–P–O bonds in [PO_4_] clusters. The broad peak located at 554.4 cm^−1^ arises from the asymmetric stretch of P–O–P bonds, while the peak centered at 406.1 cm^−1^ corresponds to the symmetric bending vibration modes related to [PO_4_] clusters (Botelho et al., 2015). For pure MoS_2_, characteristic Raman shifts located at 375.8 and 402.1 cm^−1^ are assigned to the E_2g_ and A_1g_ modes, while the peak appearing at 446.2 cm^−1^ is suggested to come from the interaction of the longitudinal acoustic phonon and Raman inactive A_2u_ modes (Koroteev et al., 2011; Lukowski et al., 2013). All characteristic peaks of both Ag_3_PO_4_ and MoS_2_ were detected in Raman spectrum of the Ag_3_PO_4_/MoS_2_ composite AM-200, indicating a complete hybridization of Ag_3_PO_4_ with MoS_2_.

**Figure 3:**
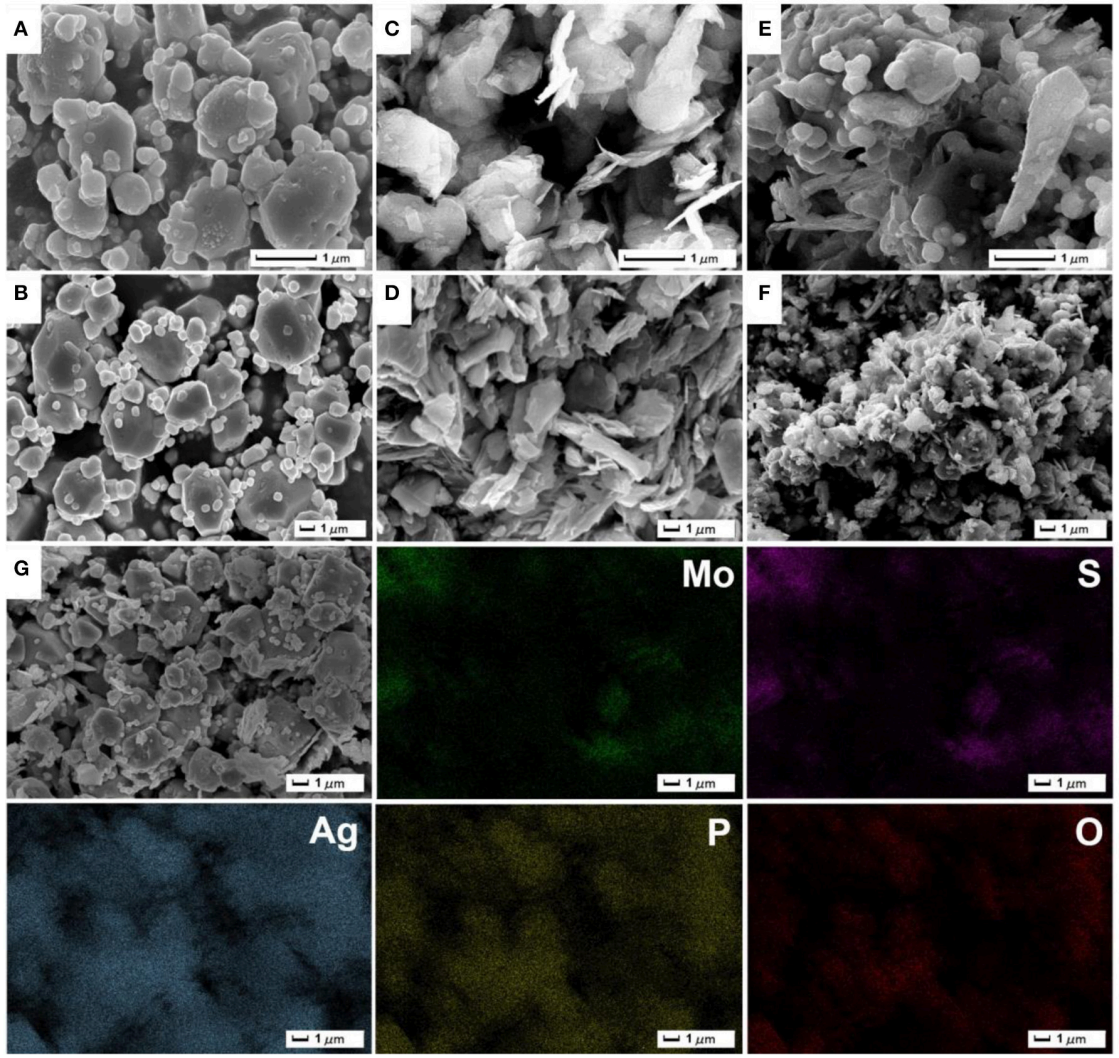
Low-magnification **(A,C,E)** and high-magnification **(B,D,F)** SEM images of pure Ag_3_PO_4_ **(A,B)**, MoS_2_ **(C,D)** as well as the composite AM-200 **(E,F)**; EDS element mapping images of AM-200 **(G)**.

The morphologies of as-prepared samples were recorded by SEM images (Figure 3). From Figures 3A,B, as-synthesized Ag_3_PO_4_ products present an irregular polyhedron structure with the size of about 1–3 μm, and a few of small particles are distributed around large particles. SEM images of ultrasonic-treated MoS_2_ material (Figures 3C,D) shows that stripped MoS_2_ exhibit a thin sheet-like nanostructure. The stripped MoS_2_ layer-like material has a certain accumulation and parts of MoS_2_ materials have not been stripped into pieces. The Ag_3_PO_4_/MoS_2_ composite AM-200 was synthesized by the *in-situ* deposition of Ag_3_PO_4_ nanoparticles on the surface of MoS_2_ nanosheets via electrostatically driven self-assembly. As displayed in Figures 3E,F, the Ag_3_PO_4_ particles are uniformly distributed on the MoS_2_ nanosheets without obvious agglomeration. It can be observed that the particle size of Ag_3_PO_4_ in AM-200 decreases slightly and is more uniform when a certain amount of MoS_2_ were hybridized with Ag_3_PO_4_, suggesting that the addition of MoS_2_ nanosheets have an effect on the particle size of Ag_3_PO_4_. The EDS element mapping images of AM-200 suggest that Mo, S, Ag, P, and O elements are homogeneously distributed, confirming the complete hybridization of Ag_3_PO_4_ particles and MoS_2_ nanosheets.

**Figure 4:**
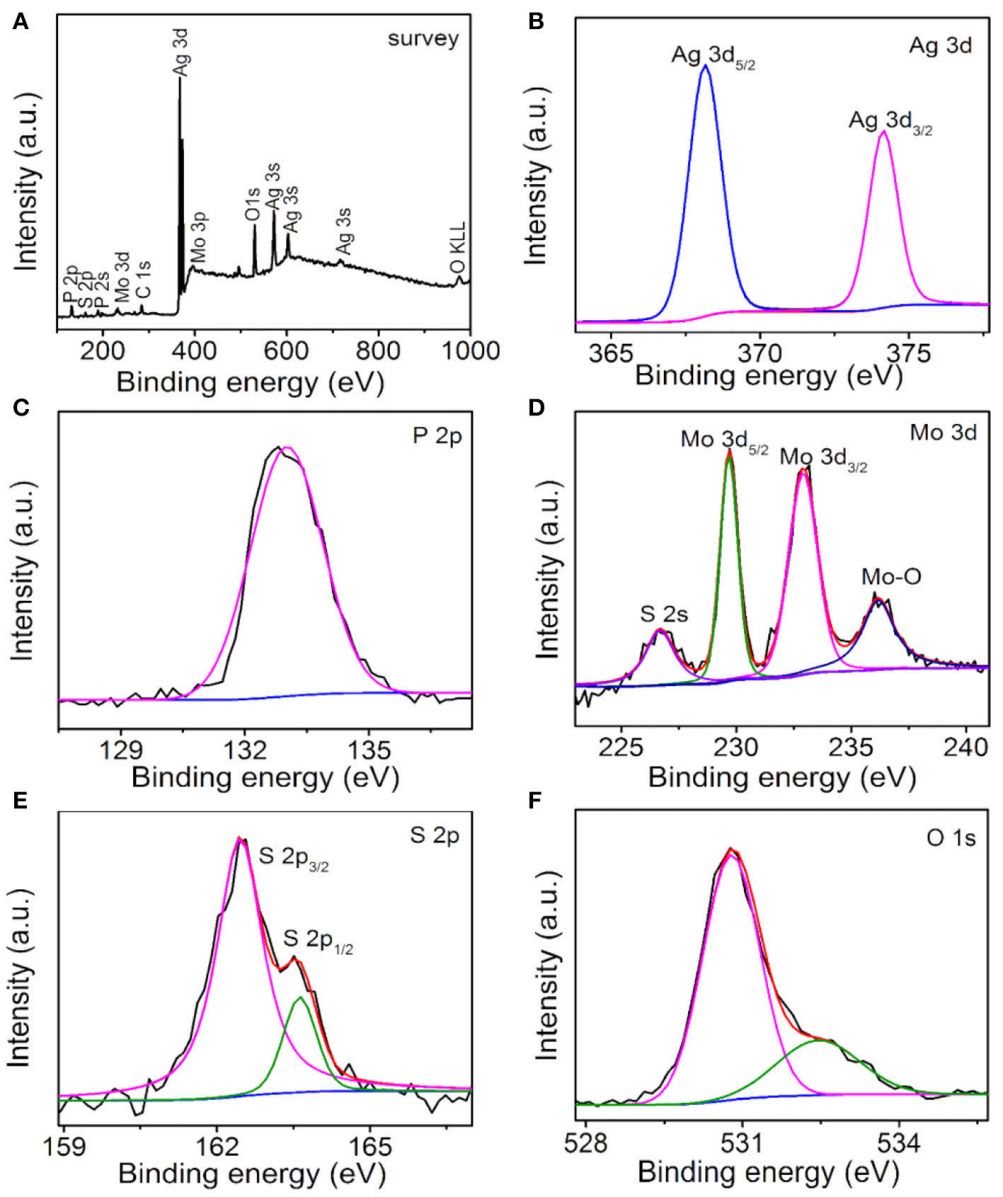
XPS spectra of AM-200: Survey **(A)**, Ag 3d **(B)**, P 2p **(C)**, Mo 3d **(D)**, S 2p **(E)**, and O 1s **(F)**.

The surface chemical compositions and states of the Ag_3_PO_4_/MoS_2_ composite AM-200 were investigated by XPS characterization, the results are shown in Figure 4. Ag, P, O, Mo, S, and C elements can be detected in the survey spectrum (Figure 4A) of as-prepared composite AM-200. The existence of C 1s peak is may due to the adventitious carbon on the surface of sample. In the high resolution spectrum of Ag 3d (Figure 4B), two peaks at 368.1 and 374.2 eV can be assigned to the Ag 3d_5/2_ and Ag 3d_3/2_, respectively. The broad peak in the P 2p spectrum (Figure 4C) located at 133.0 eV originates from the P^5+^ in the Ag_3_PO_4_. The high resolution spectrum of Mo 3d is displayed in Figure 4D, the peaks at 226.7, 229.7, 232.9, and 236.2 eV can be ascribed to the S 2s, Mo 3d_5/2_, Mo 3d_3/2_, and Mo-O binding, respectively. Particularly, the first three binding energies indicated that S and Mo elements in the MoS_2_ are found in the form of S^2−^ and Mo^4+^, respectively, and the last one might result from the exposed Mo atoms during the exfoliation process combining with the O of Ag_3_PO_4_ (Wan et al., 2017). The S 2p XPS spectrum (Figure 4E) can be divided into two peaks centered at 162.5 and 163.6 eV, respectively, corresponding to the S 2p_3/2_ and S 2p_1/2_ in the MoS_2_. The spectrum of O 1s (Figure 4F) can be fitted into two peaks located at 530.8 and 532.5 eV, originating from the O^2−^ in the Ag_3_PO_4_ and the hydroxyl group, respectively.

**Figure 5:**
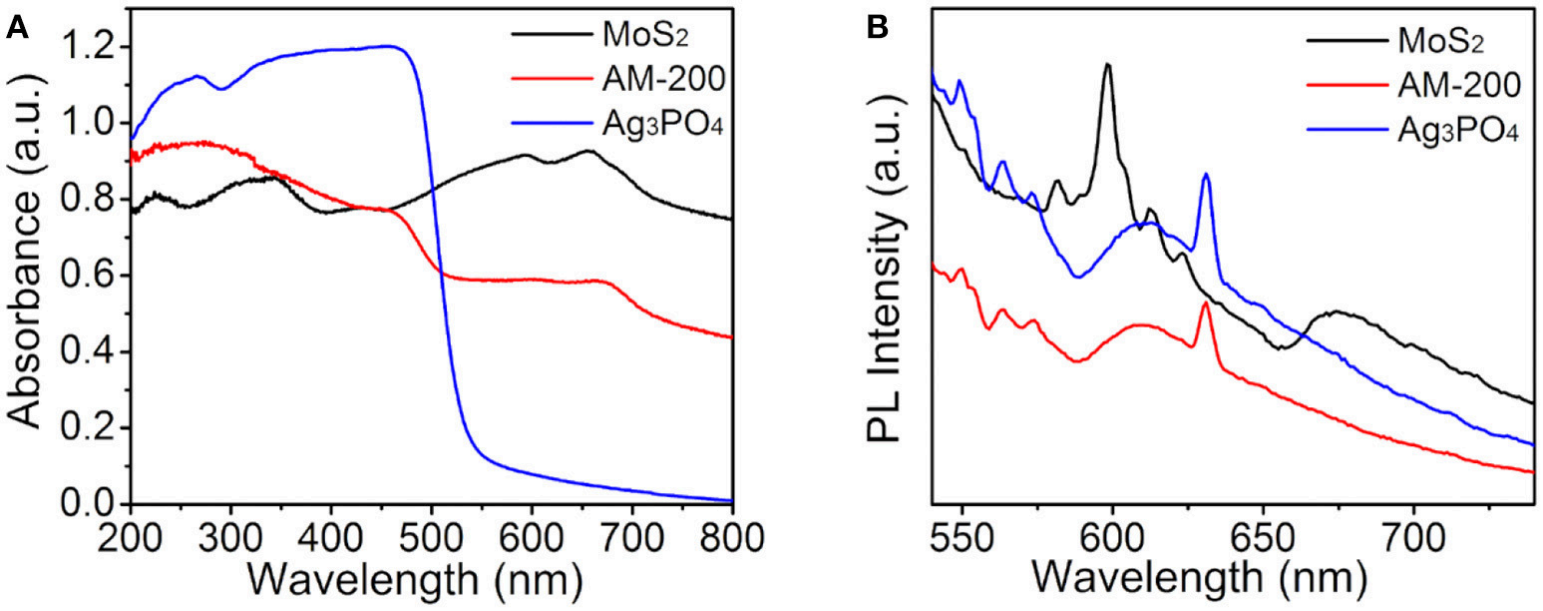
UV-vis DRS **(A)** and PL spectra **(B)** of pure Ag_3_PO_4_, MoS_2_ and the composite AM-200.

It is well-known that the utilization of visible light is one of key factors affecting the activity of a photocatalyst, therefore, the light-harvesting properties of all samples were measured by UV-vis DRS ranging from 200 to 800 nm and the absorption spectra are presented in Figure 5A. It can be seen that pure Ag_3_PO_4_ has a clear absorption edge around 530 nm, and black MoS_2_ material reveals full-spectrum absorption in the range of 200–800 nm. The light absorption intensity of Ag_3_PO_4_/MoS_2_ composite AM-200 in the wavelength range of 500–800 nm was increased when a certain amount of MoS_2_ (200 mg) were employed to hybridize with Ag_3_PO_4_, implying that the integration of MoS_2_ with Ag_3_PO_4_ favors a more efficient utilization of visible light. In addition to the light absorption, the separation of photoinduced electron-hole pairs is also believed to play a predominant role in determining the photocatalytic activity, thus the recombination of photogenerated charge carrier for the as-synthesized samples was analyzed by PL spectroscopy measurements. Figure 5B reveals Ag_3_PO_4_ has a strong excitation peak around 630 nm, stemming from the recombination of electrons and holes. After the addition of MoS_2_, the Ag_3_PO_4_/MoS_2_ composite AM-200 presented a similar position of excitation peak with Ag_3_PO_4_, and the PL emission intensity of AM-200 was weaker than those of pure MoS_2_ and Ag_3_PO_4_, suggesting that the recombination efficiency of photoexcited charge carriers in the Ag_3_PO_4_/MoS_2_ composite AM-200 has been effectively suppressed when the heterostructured composite was formed. A slower recombination rate of photogenerated electron-hole pairs boosts the enhancement in the photocatalytic performance.

**Figure 6:**
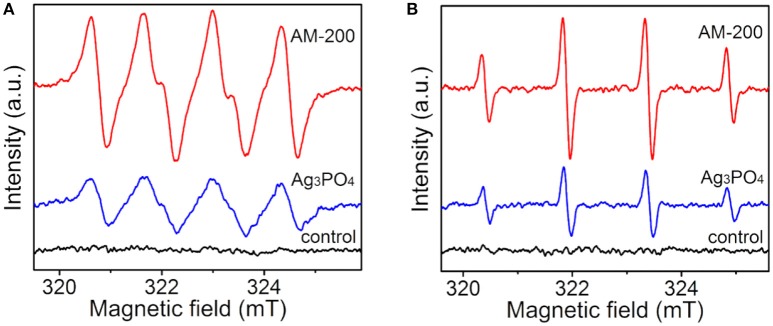
ESR spectra of radical adducts trapped by DMPO in methanol **(A)** and aqueous **(B)** dispersions of Ag_3_PO_4_ and AM-200 under light irradiation.

To further determine the influence of the redox capacity of samples on the photocatalytic activity, as well as to investigate the mechanism behind the enhanced photocatalytic oxygen evolution from water splitting, the ESR measurement was carried out to confirm active radicals *in-situ* formed under light illumination. It can be observed in Figure 6A that no obvious peak was detected in dark. Under illumination, several strong peaks arising from DMPO-captured radicals can be detected in methanol dispersion for both pure Ag_3_PO_4_ and the Ag_3_PO_4_/MoS_2_ composite AM-200, typical peaks are assigned to the spin adducts (DMPO-${\text{O}}_{2}^{•-}$). Compared with signals derived from pure Ag_3_PO_4_, higher signal intensities of DMPO-captured superoxide radicals were observed in the methanol dispersion of AM-200. It is shown in Figure 6B that no radical signal was detected in dark. A typical intensity ratio of 1:2:2:1 was determined from the spin adducts in aqueous dispersions of both Ag_3_PO_4_ and AM-200 under light irradiation, representing the generation of the spin adducts (DMPO-•OH). Similarly, the intensity of radical signal for AM-200 increased largely comparable to that of Ag_3_PO_4_.On the basis of the above results, it is concluded that higher intensities of both photo-induced ${\text{O}}_{2}^{•-}$ and •OH were recorded when a proper amount of MoS_2_ was employed.

Furthermore, the band edge positions of valence band (VB) and conduction band (CB) also have an important effect on the redox catalytic capability, which can be deduced by the following formula (Li et al., 2016):

$$\begin{array}{l} {\text{E}}_{\text{VB}}=\chi -{\text{E}}_{\text{e}}+{0.5\text{E}}_{\text{g}}\\ {\text{E}}_{\text{CB}}={\text{E}}_{\text{VB}}-{\text{E}}_{\text{g}} \end{array}$$

Where E_CB_ and E_VB_ represent the CB and VB edge potentials, respectively; χ is the electro-negativity of the semiconductor, which is the geometric mean of the electro-negativities of the constituent atoms, and χ-values for Ag_3_PO_4_ and MoS_2_ are 5.96 and 5.32 eV (Wan et al., 2017), respectively. E_e_ is about 4.5 eV, representing the free electron energy on the hydrogen scale. E_g_ was the band gap energy of the semiconductor, and E_g_-values for Ag_3_PO_4_ and MoS_2_ are about 2.45 and 1.9 eV (Yang et al., 2015c; Li et al., 2016), respectively. According to the calculation, the E_VB_-values of Ag_3_PO_4_ and MoS_2_ are about 2.69 and 1.77 eV, the top of which for both Ag_3_PO_4_ and MoS_2_ is more positive than the redox potential of O_2_/H_2_O (1.23 eV) (Xie et al., 2013), theoretically both two semiconductors are able to split water into oxygen. However, overpotential is generally required for practical water splitting, in this study, Ag_3_PO_4_ acts as the oxygen-evolving catalyst for solar-driven water splitting due to its more positive potential higher than that for water oxidation. Subsequently E_CB_ positions of Ag_3_PO_4_ and MoS_2_ are determined to be 0.24 and −0.13 eV, respectively. Therefore, the enhanced photocatalytic oxygen-generating performance over the Ag_3_PO_4_/MoS_2_ composite photocatalyst could be explained as follows: first, the electrons in the VB of Ag_3_PO_4_ could be initially excited into CB, and subsequently, may recombine with the holes in the VB of MoS_2_ via a possible Z-scheme configuration. Thus, more efficient electron-hole separations and charge transporatation occur in the illuminated hybrid materials due to the existence of highly conductive MoS_2_ sheets and possible Z-scheme pathway for electron transfer. The electron-hole recombination on the surface of Ag_3_PO_4_ can be suppressed, as a result, active holes left on the VB position of Ag_3_PO_4_ may oxidize water into oxygen effectively, leading to highly efficient oxygen evolution performance over the Ag_3_PO_4_/MoS_2_ composite photocatalysts.

## Conclusions

In conclusion, effective Ag_3_PO_4_/MoS_2_ composite photocatalysts were successfully fabricated by combining ion-exchange process and electrostatic assembly of Ag_3_PO_4_ nanoparticles on the surface of MoS_2_ nanosheets. The Ag_3_PO_4_/MoS_2_ hybrid materials demonstrated superior interfacial contact and wide-spectrum light-harvesting property in the visible light region. When employed as the catalyst for photocatalytic water splitting, it exhibited highly improved oxygen evolution performance than bulk Ag_3_PO_4_ under LED irradiation. The oxygen-evolving rate of the optimal Ag_3_PO_4_/MoS_2_ composite (AM-200) is nearly five times faster than pure Ag_3_PO_4_. The combined characterizations and theoretical analysis on band structures suggest that the enhanced water oxidation efficiency is attributed to remarkable response to visible light, more efficient charge transportation and possibly specific Z-scheme pathway derived from matched band positions. The finding in this work offers a great opportunity in designing and synthesizing novel composite photocatalytic materials for applications in solar energy conversion, allowing us to develop an understanding of the fundamental mechanisms of Ag_3_PO_4_-based composite photocatalytic materials.

## Author contributions

XY and QL: designed the project, guided the study, and polished the manuscript; XC, XX, and LT: conducted the experiments and characterized the samples; HT: revised the manuscript.

### Conflict of interest statement

The authors declare that the research was conducted in the absence of any commercial or financial relationships that could be construed as a potential conflict of interest.
